# Clinical outcomes of femoral shaft non-union: dual plating versus exchange nailing with augmentation plating

**DOI:** 10.1186/s13018-018-1002-z

**Published:** 2018-11-20

**Authors:** Wei Zhang, Zhuo Zhang, Jiantao Li, Licheng Zhang, Hua Chen, Peifu Tang

**Affiliations:** Department of Orthopedic Surgery, General Hospital of Chinese People’s Liberation Army, 28 Fu-Xing Road, Beijing, 100853 People’s Republic of China

**Keywords:** Femoral non-union, Exchange nailing, Augmentation plating, Dual plating

## Abstract

**Background:**

By comparing clinical outcomes between dual plating (DP) and exchange nailing with augmentation plating (EN/AP), we aimed to provide better treatment strategies for femoral shaft non-union.

**Methods:**

We retrospectively reviewed 30 patients with aseptic femoral shaft non-union at our level 1 trauma center between January 2014 and January 2017. All patients underwent a one-stage, definitive revision procedure, including DP for 16 patients and EN/AP for 14 patients. Perioperative surgical trauma, fracture healing, complications, and the time to return to work were evaluated.

**Results:**

Twenty-nine patients achieved fracture healing. In the EN/AP group, the fracture healing rate was 100%, the healing time was 5.7 ± 1.7 months, and the time of return to work was 8.2 ± 2.9 months. In the DP group, the fracture healing rate was 94%, the healing time was 8.4 ± 4.1 months, and the time of return to work was 18.4 ± 10.3 months. In terms of fracture healing and return to work, the patients in the EN/AP group required less time than those in the DP group, and the differences were statistically significant (*p* = 0.024 and *p* < 0.01 respectively). Except for the length of the incision, the two groups showed no statistically significant differences in operative time, postoperative deformity, and complications.

**Conclusions:**

Both EN/AP and DP are important surgical options for femoral shaft non-union. Compared to DP, EN/AP resulted in a shorter incision, faster fracture healing, and a shorter time to return to work.

**Trial registration:**

ChiCTR-ORC-17014062

## Introduction

Mechanical stability improvement is the basis for treatment selection as well as the key to success in femoral shaft non-union cases [1–4]. Nail dynamization, exchange lateral plating (EP), and exchange nailing (EN) have been generally applied in clinical practice [5–9]. However, due to the mechanical instability of revision procedures mentioned above, the failure rate is as high as 20–50% [7, 10–13]. Optimal mechanical stability and deformity correction can be achieved during exposure of the non-union site in the procedures of dual plating (DP) and exchange nailing with augmentation plating (EN/AP) [14–18]. Evidence indicates that most patients who received either surgical procedure achieved fracture healing.

To avoid massive surgical trauma and complications, improve fracture healing and accelerate rehabilitation, evaluating the advantages and disadvantages of DP and EN/AP is critical. To our knowledge, no comparative study concerning both surgical procedures has been published.

Therefore, we retrospectively reviewed the medical data of patients with femoral shaft non-union who had been treated via DP or EN/AP in our trauma center for the first time. Surgical trauma, complications, fracture healing, and time to return to work were analyzed to evaluate the advantages and disadvantages of the two surgical procedures.

## Materials and methods

### Inclusion and exclusion criteria

This study is a retrospective, observational study. Its design and implementation conformed with the Declaration of Helsinki and were approved by the ethics committee of our hospital. A total of 63 patients with aseptic femoral shaft non-union who underwent surgery between January 2014 and January 2017 were retrospectively analyzed. Non-union was diagnosed based on a fracture that did not achieve union within 9 months after surgery and did not show any signs of healing for 3 consecutive months. According to the AO/OTA Classification of Fractures and Dislocations, the femoral shaft refers to the segment from the proximal end at the lower level of the lesser trochanter to the distal end at the square edge, with the femoral intercondylar width reflecting the sides. The inclusion criteria were (1) age > 18 years old; (2) bone defects < 6 cm; and (3) treatment via DP or EN/AP. The exclusion criteria were (1) infectious non-union; (2) pathological non-union; (3) patients with neurological diseases affecting the motor nerves; and (4) patients with mental illness who were unable to cooperate with treatment.

**Fig. 1:**
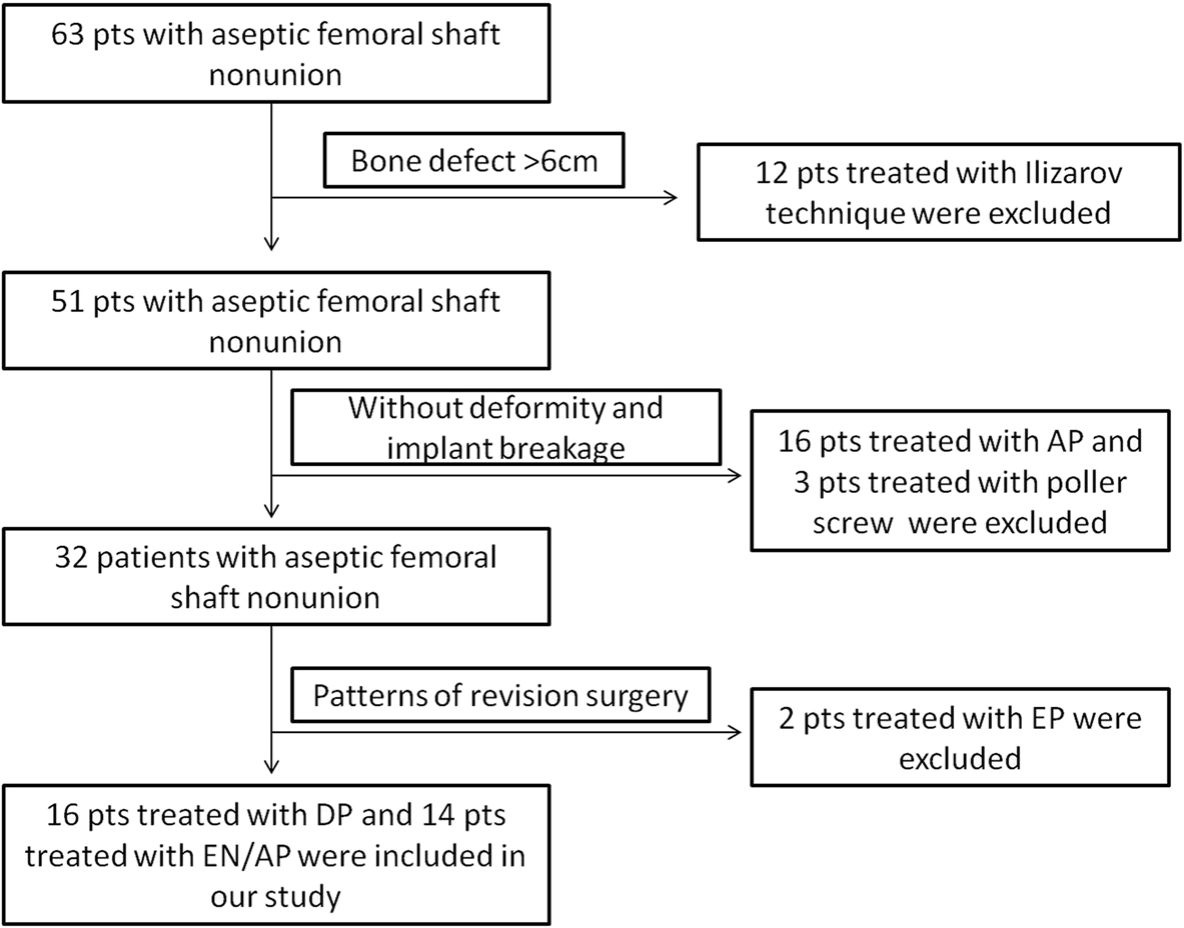
The algorithm for patients included and excluded

Of the 30 included patients, 14 were included in the EN/AP group and 16 were included in the DP group (see Fig. 1). In the EN/AP group, 7 patients showed previous implant breakage (5 intramedullary nails (IMNs) and 2 plates), 11 patients showed severe deformities, including 6 cases of leg length discrepancy (LLD) (range, 2–3 cm), 9 cases of angular deformities (range, 7°–30°) and 8 cases of rotational deformities (5 with external rotation in the range of 30°–40° and 3 with internal rotation in the range of 20°–30°), and 4 patients had bone defects, including 3 with a medial bone defect and 1 with a segmental defect (3 cm). In the DP group, 11 patients showed previous implant breakage (5 IMNs and 6 plates), 15 patients showed severe deformities, including 4 cases of LLD (range, 2–3 cm), 13 cases of angular deformity (range, 10°–25°) and 10 cases of rotational deformities (7 with external rotation in the range of 15°–30° and 3 with internal rotation in the range of 15°–20°), and 5 patients showed bone defects, including 2 with a medial bone defect and 3 with a segmental defect (range, 2–3 cm) (see Table 1).

**Table 1:** Demographic data of the patients preoperatively

Characteristic	EN + AP group (*n* = 14)	DP group (*n* = 16)	*p* value
Mean age (years)	38.8 ± 16.0 (19–71)	40.9 ± 11.7 (23–68)	0.684
Sex (M:F)	9:5	13:3	0.295
Number of previous surgeries			0.196
1	11	9	
≥ 2	3	7	
Time since fracture (months)	32.3 ± 42.3 (9–170)	31.6 ± 38.0 (9–170)	0.965
Previous internal fixation (*n*)			0.151
Nailing	12	10	
Plating	2	6	
Breakage of internal fixation (*n*)	7	11	0.296
Non-union types (*n*)			0.79
Hypertrophic	4	6	
Oligotrophic	6	5	
Atrophic	4	5	
Deformities (*n*)			
LLD	6	4	0.301
Angulation	9	13	0.295
Rotation	8	10	0.765
Bone defect (*n*)	4	5	0.873

### Surgical technique

All patients underwent a one-stage, definitive revision procedure. According to the full-length radiograph and CT scan of both lower extremities, the degree of deformity and the length of the bone defect were clearly determined. The contralateral femur length, femoral neck anteversion, and neck-shaft angle served as the references for deformity correction.

For the patients in the DP group, the non-union site was fully exposed. Before removing the previous implant, a 2.0-mm short Kirschner wire was fixed at each side of the non-union site along the long axis of the femur in parallel as the reference for correction of the rotational deformity. Then, the lateral plate was first fixed to the proximal femur, the distal part of the femur was rotated and retracted, the length of the gap between the sites was measured, and then the distal end was fixed. Finally, fixation of the anteromedial plate was completed.

For the patients in the EN/AP group, after the correct entry point of the greater trochanter was determined, a new nail was inserted to maintain correct alignment. The proximal locking screw was fixed, and the distal part was rotated and pulled with a retractor to restore the length and rotational alignment. Next, distal interlocking screws were fixed. Finally, the augmentation plate was placed.

All patients received autologous bone grafting utilizing the iliac crest, except for patients with a hypertrophic non-union without bone defects.

### Postoperative rehabilitation and statistical analysis

Postoperatively, all patients underwent CT scans with 3D reconstruction to evaluate residual deformities. Meanwhile, they were allowed to perform non-weight-bearing exercise immediately to avoid knee stiffness. 1 month after surgery, the patients were permitted to engage in partial weight-bearing with a crutch for protection. After fracture healing was confirmed, the patients were allowed to initiate weight-bearing activities without a crutch. To confirm fracture healing, both clinical and radiological criteria should be met at the same time; that is, full weight-bearing on the affected limb can be achieved without pain, X-rays show a fuzzy fracture line, and callus continuity can be observed on three sides of the bone cortex. If fracture healing could not be confirmed by plain film, a CT scan with 3D reconstruction was performed. The criteria for healing based on CT exam were the fuzzy fracture line and callus continuity on more than 25% of the coronal plane [19]. Radiological consolidation was evaluated by two surgeons.

Perioperative trauma [operative time (min), incision length (cm)], fracture healing, postoperative complications and the corresponding treatments, and the time to return to work (months) were recorded and analyzed. The primary outcomes included the fracture healing rate, healing time, and the time to return to work, and the others were considered as the secondary outcomes. SPSS 22.0 statistical software was used for the analysis. Measurement data were expressed as the mean ± standard deviation. Normal distribution of data was tested in advance. *t* tests were selected for normal distributed data. Otherwise, Mann-Whitney *U* tests were applied. Count data were compared using chi-square tests. The significance level of the analysis was α = 0.05.

## Results

All patients were followed up, with a median follow-up of 2 years (range, 1–4 years). No statistically significant difference in the follow-up period (2.59 ± 1.16 years vs 2.03 ± 1.2 years, *p* = 0.206) was observed between the DP and EN/AP groups. Except for incision length (*p* = 0.000 < 0.05), no significant differences were noted in terms of the operative time and postoperative deformity between the two groups (see Table 2).

**Table 2:** Perioperative data

Variable	EN + AP group	DP group	*p* value
Length of the incision (cm)	19.3 ± 5.8	30.4 ± 5.9	0.000
Operative time (min)	194.0 ± 33.0	182.0 ± 42.0	0.407
Deformities (*n*)			
LLD	1	0	0.277
Angulation	1	0	0.277
Rotation	4	2	0.272

**Fig. 2:**
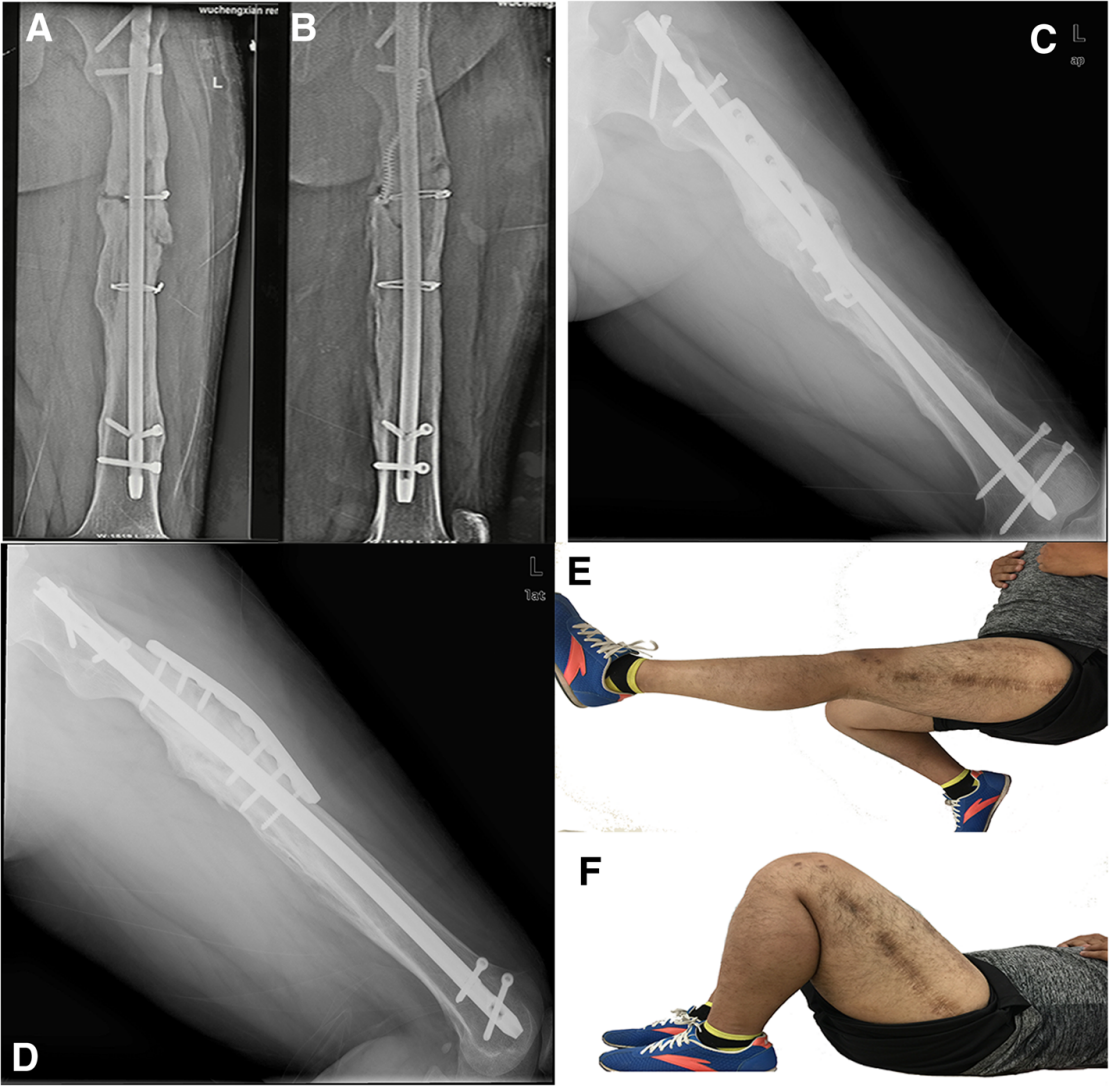
A 26-year-old male with left femoral shaft non-union after nailing. **a**, **b** X-ray showing breakage of the distal locking screw with obvious varus deformity and a bone defect (3 cm) preoperatively. **c**, **d** X-ray showing fracture healing 6 months after EN/AP. **e**, **f** The knee joint function of the affected side was good

**Fig. 3:**
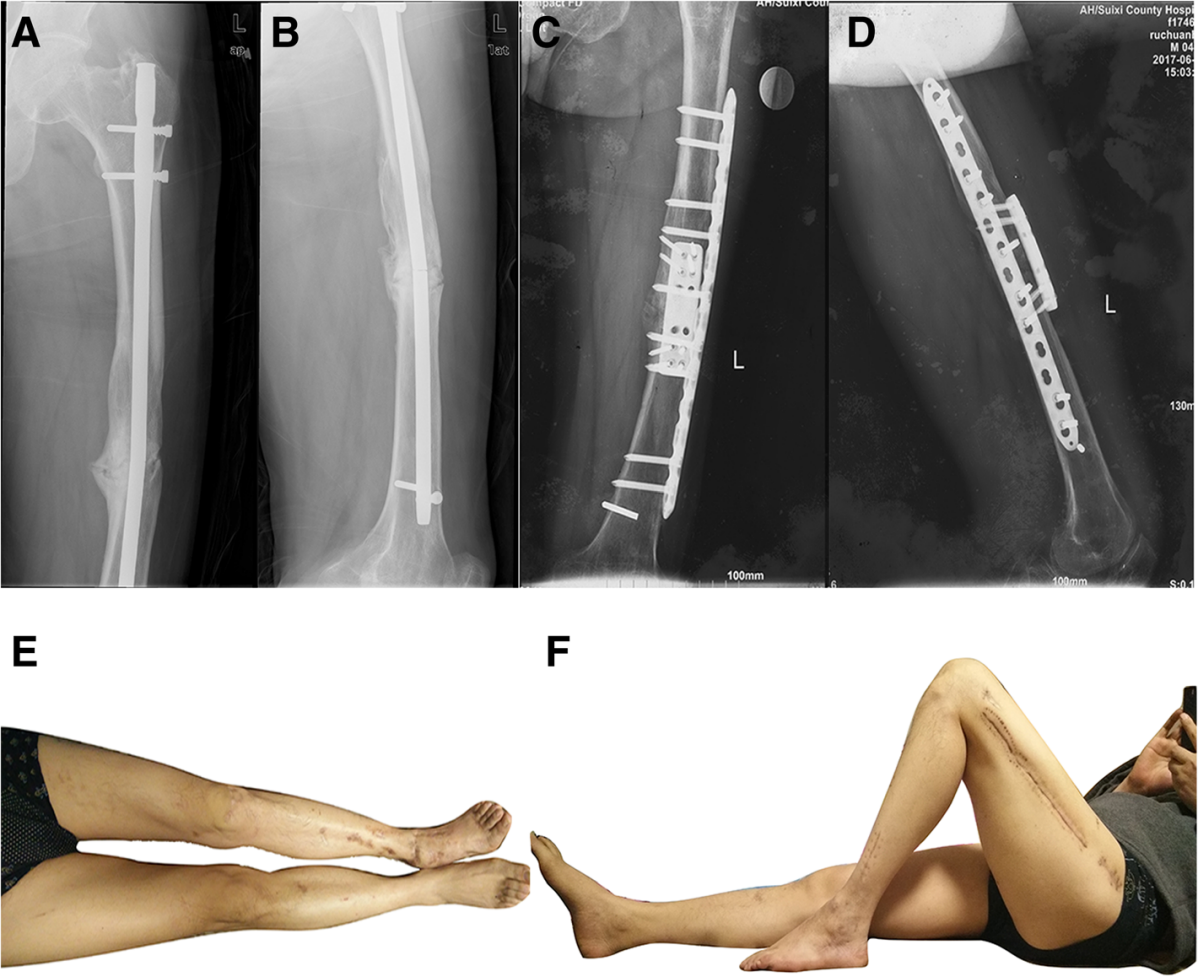
A 43-year-old male with left femoral shaft non-union after nailing. **a**, **b** X-ray showing breakage of the intramedullary nail and distal locking screw with obvious internal rotation and varus deformity preoperatively. **c**, **d** X-ray showing fracture healing 7 months after DP. **e**, **f** The knee joint function of the affected side was good

In the EN/AP group, the fracture healing rate was 100%, and the fracture healing time was 5.7 ± 1.7 months. In the DP group, the fracture healing rate was 94%, and the fracture healing time was 8.4 ± 4.1 months. The healing time and time to return to work were shorter in EN/AP group, with statistically significance (*p* = 0.024 in healing time, and *p* < 0.01 in time to return to work) (see Table 3). In the DP group, two patients did not return to work because one patient was followed up for only 1 year, while the other patient still exhibited non-union. Classic cases from the two groups are shown in Figs. 2 and 3.

**Table 3:** Postoperative outcomes and complications

Outcome	EN + AP group (*n* = 14)	DP group (*n* = 16)	*p* value
Number of unions	14	15	0.341
Time to union (months)	5.7 ± 1.7	8.4 ± 4.1	0.024
Return to work (months)	8.2 ± 2.9	18.4 ± 10.3	< 0.01
Number of complications	6	10	0.153
Superficial infection	0	1	
Bone infection	0	0	
Knee stiffness	2	4	
Refracture	0	1	
Non-union	0	1	
Malunion	4	2	
Reoperation	0	1

In terms of complications, no significant difference was found between the two groups. In the EN/AP group, all patients with complications refused additional surgical procedures because they were basically satisfied with their function. In the DP group, one patient had a superficial soft tissue infection at 1 month postoperatively; therefore, debridement and vacuum-assisted closure were performed. After the infection was controlled, wound closure was carried out again. Additionally, intravenous antibiotics were administered for 3 weeks based on drug sensitivity testing. One patient showed no fracture healing 14 months after the operation and refused to undergo bone grafting again; this patient has been followed up for 2 years, and the fracture still has not healed. One patient received implant removal surgery at a local hospital 2 years postoperatively, and refracture occurred 3 months later. Subsequently, the patient underwent plating fixation and bone grafting in our hospital again, and the fracture healed at 12 months after this operation. Patients exhibiting knee stiffness and malunion did not receive additional surgical treatment (see Table 3).

## Discussion

The primary objective of femoral shaft non-union treatment is improved mechanical stability. The anti-rotation stability of previous nailing is reduced after dynamization procedures, leading to an unacceptable incidence of failure up to 40–60% [12, 13]. The intractability of rotational and bending instability remains unresolved, even when a larger nail is used. Up to 20~50% of patients who underwent EN did not achieve fracture healing [10, 20]. However, EN/AP is not only minimally invasive and can achieve axial mechanical stability via nailing but can also solve the problem of insufficient anti-rotation and anti-angulation stability with nailing (especially non-isthmus fractures). On the other hand, compared to EP, DP can solve the problem of eccentric fixation, build a more mechanically stable construct, and stabilize the autologous bone grafts around the non-union site [4, 21]. Therefore, DP and EN/AP, as three-dimensional fixation constructs that can provide sufficient multiplanar stability, are suitable for patients with femoral shaft non-union.

Publications show that the healing rate for femoral shaft non-union is as high as 90% [14–18]. In our study, 29 patients (96%) finally achieved fracture healing, which is similar to previous reports. The fracture healing time with DP has been reported to be 5.2–6 months [14–16], which is dramatically shorter than that with EN/AP (6–13 months) [17, 18] in previous reports. However, different results were observed in our study for the both healing time and the time to return to work, which were significantly shorter with EN/AP versus DP. Multiple factors are considered to be related to these results. First, excessive periosteal dissection is the most important reason for the prolonged fracture healing in the DP group [14]. Second, for patients in the EN/AP group, intramedullary debris can infiltrate the non-union site to achieve “autologous bone grafting” by reaming [8, 9]. Meanwhile, reaming can also increase the periosteal blood supply and stimulate callus formation [8, 9]. Finally, the fracture may heal faster due to less periosteal soft tissue detachment and better restoration of the endosteal blood supply [22].

Deformity correction is the main surgical technical difficulty in femoral non-union cases. Plating has been the main method of deformity correction for a long time [4, 14, 23]. With direct exposure of the non-union site, debridement, surface contact, deformity correction, and rigid fixation can be achieved simultaneously [23]. However, IMN has obvious shortcomings in the correction of rotation and angle deformity. Meanwhile, an augmentation plate with nailing in situ cannot correct deformities [24, 25]. Therefore, some surgeons have proposed the application of exchange nailing with augmentation plating (EN/AP) for the treatment of femoral shaft non-union with severe deformities. Wang et al. [17] described 12 patients (9 cases of shortening deformity and 3 cases of rotational deformity) with femoral shaft non-union who underwent the EN/AP procedure and achieved effective correction. Yang et al. [18] reported the application of EN/AP in two patients with severe deformities and obtained good results. In our study, 11 patients had severe deformities and underwent EN/AP. The EN/AP procedure achieved excellent deformity correction through limited exposure of the non-union site, which showed similarly good clinical results with those of DP, as reflected by the shorter incision length.

However, this study was a retrospective study with a relatively small sample. The advantages and disadvantages of both procedures require further investigation with a larger sample in well-designed clinical studies. Furthermore, some patients did not attend the scheduled appointment for the follow-up and radiological examinations, so the data might not truly reflect the healing time. More rigorous follow-up and more convenient diagnostic techniques are needed to improve the data accuracy. Finally, the choice of surgical techniques depends on the patient’s conditions and surgeon’s preference, the bias in treatment selection also is our trial limitation.

## Conclusions

Overall, both EN/AP and DP can provide excellent mechanical stability to the non-union site and achieve effective deformity correction. Compared to the DP group, the EN/AP group showed a shorter incision length, faster fracture healing, and a shorter time to return to work. However, no significant differences were observed in terms of surgical trauma and complications in our study.

## Abbreviations

AP: Augmentation plating
DP: Dual plating
EN/AP: Exchange nailing with augmentation plating
EP: Exchange lateral plating
IMN: Intramedullary nail
LLD: Leg length discrepancy
pts: Patients
